# P-2130. Low-Level CMV Viremia Predicts Increased Risk of CMV Disease and Subsequent High-Level Viremia in Lung Transplant Recipients: A Multicenter EHR-Based Study

**DOI:** 10.1093/ofid/ofaf695.2294

**Published:** 2026-01-11

**Authors:** German Contreras, George Golovko

**Affiliations:** University of Texas Medical Branch, Houston, Texas; University of Texas Medical Branch at Galveston, galveston, Texas

## Abstract

**Background:**

The clinical significance of low-level cytomegalovirus (CMV) viremia (137–999 IU/mL) in lung transplant recipients remains uncertain. We evaluated whether subthreshold CMV replication predicts progression to CMV disease or high-level viremia (≥1,000 IU/mL) within one year post-transplant.

**Methods:**

We performed a retrospective cohort study using the TriNetX Research Network, a federated health research platform aggregating real-world EHR data from 101 healthcare organizations. Adult lung transplant recipients without prior CMV disease were categorized based on CMV PCR viral load into two groups: low-level viremia (137–999 IU/mL) and minimal viremia (< 137 IU/mL). Following 1:1 propensity score matching based on demographics and clinical characteristics, 142 patients were included per cohort. Outcomes assessed over a 6-month window included CMV disease and subsequent high-level viremia (≥1,000 IU/mL).

**Results:**

At 6 months, CMV disease occurred in 42.3% of the low-level viremia group versus 30.3% in the minimal viremia group (Risk Difference 12.0%, 95% CI 0.9–23.1, p=0.036; Risk Ratio 1.40). Kaplan-Meier survival analysis showed a significantly higher hazard for CMV disease in the low-level group (HR 1.55, 95% CI 1.05–2.30, p=0.026).

Similarly, high-level CMV viremia developed in 19.7% of low-level patients versus 10.6% of minimal viremia patients (Risk Difference 9.2%, 95% CI 0.9–17.4, p=0.031; Risk Ratio 1.87), with a higher hazard of progression (HR 1.92, 95% CI 1.03–3.60, p=0.038).

**Conclusion:**

Low-level CMV viremia was associated with a significantly higher risk of both CMV disease and high-level viremia within six months post-transplant. These findings suggest that even subthreshold CMV replication warrants closer surveillance and may inform earlier intervention strategies in lung transplant recipients.

**Disclosures:**

All Authors: No reported disclosures

